# Teleophthalmology and retina: a review of current tools, pathways and services

**DOI:** 10.1186/s40942-023-00502-8

**Published:** 2023-12-05

**Authors:** Jonathan Than, Peng Y. Sim, Danson Muttuvelu, Daniel Ferraz, Victor Koh, Swan Kang, Josef Huemer

**Affiliations:** 1 Moorfields Eye Hospital NHS Foundation Trust, 162 City Road, London, UK; 2 Department of Ophthalmology, Rigshospitalet, Copenhagen University Hospital, Copenhagen, Denmark; 3 MitØje ApS/Danske Speciallaeger Aps, Aarhus, Denmark; 4 D’Or Institute for Research and Education (IDOR), São Paulo, Brazil; 5 Department of Ophthalmology, National University Hospital, Singapore, Singapore; 6 Institute of Ophthalmology, University College London, London, UK; 7 Department of Ophthalmology and Optometry, Kepler University Hospital, Johannes Kepler University, Linz, Austria

## Abstract

Telemedicine, the use of telecommunication and information technology to deliver healthcare remotely, has evolved beyond recognition since its inception in the 1970s. Advances in telecommunication infrastructure, the advent of the Internet, exponential growth in computing power and associated computer-aided diagnosis, and medical imaging developments have created an environment where telemedicine is more accessible and capable than ever before, particularly in the field of ophthalmology. Ever-increasing global demand for ophthalmic services due to population growth and ageing together with insufficient supply of ophthalmologists requires new models of healthcare provision integrating telemedicine to meet present day challenges, with the recent COVID-19 pandemic providing the catalyst for the widespread adoption and acceptance of teleophthalmology. In this review we discuss the history, present and future application of telemedicine within the field of ophthalmology, and specifically retinal disease. We consider the strengths and limitations of teleophthalmology, its role in screening, community and hospital management of retinal disease, patient and clinician attitudes, and barriers to its adoption.

## Introduction

Telemedicine is the use of telecommunication and information technology for the purpose of providing remote health assessments and therapeutic interventions [1]. It may be synchronous, involving real-time interaction amongst participants separated in space via communication technology, or asynchronous (“store-and-forward”), separating the collection of medical data and its review in time and space [2]. It may involve communication between healthcare providers seeking clinical guidance and support from other healthcare providers (provider-to-provider telemedicine), or between remote healthcare users seeking health services and healthcare providers (client-to-provider telemedicine) [3]. The evolution of telemedicine since its inception in the 1970s, involving landline telephones and televisions “cumbersome to move about, probably too expensive to have in every examination room, with almost one-third of consults encountering technical difficulties” [4], would render it almost unrecognisable in its present form to those early pioneers. The advent of the Internet, wireless network protocols, broadband cellular networks, and high-resolution digital photography, together with the exponential growth in computing power that has made computer-aided diagnosis (CADx) possible via artificial intelligence (AI), machine learning (ML) and its subset deep learning (DL), has created an environment where telemedicine can equal and surpass traditional face-to-face medicine in terms of patient safety, outcomes and user satisfaction, with the potential to make healthcare more equitable, accessible and efficient than ever before [5]. The recent COVID-19 pandemic has provided additional stimulus to accelerate the development and adoption of telemedicine in routine clinical practice. In no field is the potential of telemedicine more evident than ophthalmology, and in particular retinal disease—the plethora of image-based investigations in retinal clinical practice makes it uniquely suited to a telemedicine approach. This review will discuss the history, current and future application of telemedicine within ophthalmology and in particular the field of retina.

## Telemedicine in the pre-hospital setting

### Screening

The impact of telemedicine in ophthalmology is most evident in its established role worldwide in the screening of retinal disease, namely diabetic retinopathy (DR) and retinopathy of prematurity (ROP).

### Diabetic retinopathy

Diabetes mellitus (DM) affects approximately half a billion people worldwide [6], with a third of these suffering from diabetic retinopathy [7]. Amongst people over 50 years old, an estimated 861,000 people are blind and 2.95 million people moderately to severely vision impaired as a result [8]. DR is archetypal for a condition meeting the criteria set by Wilson and Jungner of a disease amenable to screening [9]; it is an important health problem, possesses a detectable asymptomatic phase, has an acceptable, safe and precise diagnostic test (retinal photography), and effective interventions exist which are more beneficial earlier in the disease process.

The creation of a standardised definition of referrable disease has been vital to the efficacy of screening programmes worldwide and their adaptability to an asynchronous telemedicine approach. The Arlie House Symposium in 1968 was the first to objectively define and classify DR through standardised fundus photography [10]. A modified form of the Arlie House Classification based on seven field 30° stereoscopic colour fundal photographs (CFP) was used in the first landmark trial to demonstrate the reduction of severe vision loss in DR through panretinal photocoagulation [11], and later, the Early Treatment Diabetic Retinopathy Study (ETDRS) used a further modified form (ETDRS grading criteria) which has been accepted as the gold standard for detection of referable disease [12]. However, the imaging requirements of the ETDRS criteria are not feasible for a screening setting due to the staff training, patient cooperation, and time required to attain stereo seven field CFP imaging in every patient. Adaptations to reduce test demands whilst maximising test sensitivity and specificity have therefore been made by screening programmes worldwide. The American Telemedicine Association (ATA) has developed four categories of validation for DR telemedicine screening programmes depending on the extent to which programmes can perform against the ETDRS gold standard (Table 1) [13]. In 1995, The British Diabetic Association put forward a consensus statement from clinicians that a screening test for referable DR should have minimum sensitivity of 80% and specificity of 95% [14]. Two field mydriatic 45° CFP (centred on the optic disc and macula) meets these criteria, with sensitivity 80.2% and specificity 96.2% [15]—the ease and speed of acquisition of this approach, together with its acceptability to patients, has made this approach the most common in screening programs worldwide.

**Table 1:** American Telehealth Association DR telehealth program categories of validation [13]

Validation category	Capability
1	Separation of patients into two categories: 1) No or minimal DR (ETDRS level ≤ 20) 2) More than minimal DR (ETDRS level > 20)
2	Separation of patients into two categories: 1) Non-sight-threatening DR 2) Sight-threatening DR (diabetic macular edema (DME), severe non-proliferative DR (ETDRS level ≥ 53), or proliferative DR (ETDRS level ≥ 61)
3	Identification of ETDRS defined levels of non-proliferative DR (mild, moderate, severe), proliferative DR (early, high-risk), and DME with sufficient accuracy to determine follow-up and treatment strategies at a level equivalent to dilated clinical retinal examination
4	Ability to match or exceed the ability of ETDRS photos to identify and determine severity of DR and DME

The first recognised DR screening programme was developed in Iceland in 1980 for patients with insulin-dependent DM [16–18]. However, this programme involves annual fundus examination and DR grading by an ophthalmologist in addition to CFP acquisition rather than a telemedicine approach. In 1990, DR screening via four field 45° CFP acquisition by mobile fundus photography teams distributed around primary and secondary healthcare centres (with subsequent analysis by ophthalmologists) was introduced in Stockholm, Sweden, which resulted in a 45% decrease in the incidence of blindness (visual acuity (VA) < 1.3 LogMAR)—one of the first examples of DR screening via telemedicine [19]. Presently, numerous telemedicine-based DR screening programmes exist worldwide. The constituent countries of the United Kingdom (UK) have the largest national programmes presently [20]—the asynchronous store-and-forward platforms of England, Scotland, Wales and Northern Ireland, involving acquisition of two field mydriatic 45° CFP by trained technicians and later grading of photos by graders and referral to hospital eye services where indicated, has been successful in reducing rates of sight loss secondary to DR such that in 2010, DR was no longer the primary cause for blindness registration in the UK for the first time in 50 years [21]. Singapore possesses the second largest national DR screening programme, with the Singapore Integrated Diabetic Retinopathy Programme (SiDRP), commenced in 2010, having screened over 600,000 diabetics in total. CFPs are captured at multiple places including polyclinics and primary care providers, and then sent to a centralised reading center where photographs are graded synchronously (within one business day at most), and a standardised referral protocol followed. Cost savings from this model, versus the previous approach of review of CFPs by family physicians within the polyclinic in which they were captured, is estimated to be $144 per person [22]. Although there is no national screening programme in the United States of America, several large validated regional programmes exist. Examples include the DR screening programme run by the Kaiser Permanente, the largest private health insurer in the US, and the Indian Health Service-Joslin Vision Network (IVS-JVN) Teleophthalmology Program, the largest ATA Category 3 program in the USA. The IVS-JVN has performed over 226,333 studies on diabetics within the American Indian and Alaska native population thus far [23–25]. Both programmes utilise asynchronous centralised reporting of CFP, two field in the case of Kaiser Permanente and five field for the JVN.

The aforementioned DR screening programmes all require significant infrastructure to enable standardised CFP acquisition at designated centres for the relevant population, with means to rapidly transfer images to reading centres. Investment in such an infrastructure may be prohibitively expensive, particularly in the developing world. Alternative methods of image acquisition utilising equipment that is highly portable, user-friendly and potentially already available to patients is therefore attractive, as it might enable access to DR screening even in resource-poor settings. Smartphone ophthalmoscopy, the use of an in-built smartphone camera to obtain retinal images, has been shown to be viable in this role—a prospective comparison performed in India demonstrated high agreement between a smartphone-based portable imaging system (Remidio Fundus on Phone) and four field CFP in detecting both any DR and sight-threatening DR in 2015 [26].

One major limitation preventing widespread adoption worldwide of DR screening programmes is the need for a large number of trained graders in reading centres to analyse the large volume of CFP images generated by screening programmes. Automated analysis of retinal images for referable diabetic retinopathy is therefore an attractive proposition as it would render this requirement redundant. The first paper in this field, published in 1973, demonstrated computer-aided detection (CADe) of retinal vascular contour lines [27]. Subsequent to CADe, digital photography and increased computing power facilitated the development of computer-aided diagnosis (CADx) systems which qualitatively detect features of DR, calculate disease probability, and risk stratify disease. Earlier CADx algorithms utilised thresholding, edge detection, processing and filters to identify disease [28], with more advanced models using ensemble-based approaches [29], multi lesion approaches or content-based image retrieval [30]. An automated retinal image analysis system (ARIAS) developed by Abràmoff et al. in 2008 and refined in 2013 demonstrated 96.8% sensitivity and 59.4% specificity in detecting referrable DR [23–25, 31, 32]. The iGradingM ARIAS is presently used in a category 1 grading (disease/no disease) role within the Scottish DR screening programme, having been extensively validated [33, 34]. It has been evaluated to have a sensitivity to detect DR of 97.8% and specificity of 41.2% [35]. Category 1 grading is labour intensive due to the high number of CFPs generated by screening programmes, and hence its automation has significant cost-saving potential—it has been estimated that automated grading saves £212,695 per 180,000 screened in Scotland [36].

Deep learning (DL), a subset of machine learning using multiple layers to progressively extract higher-level features from a raw input, usually via training of a convolutional neural network (CNN), has facilitated the creation of CADx algorithms which are rapidly superseding the previously mentioned approaches. Although the concept of DL has been present since 1967 [37], recent advances in computing power, large volumes of digital data, and publically available pre-trained CNNs have led to the current explosion in research utilising this approach in healthcare [38–42]. DL has already been shown to perform equivalently to humans in the detection of disease from medical imaging [43–47]. Abramoff et al. enhanced their previous ARIAS by integrating DL, renaming it IDx-DR, to achieve 96.8% sensitivity and 87% specificity in detecting referrable DR in 2016 [48]. IDx-DR has since been validated against multiple datasets both retrospectively [49] and prospectively on 900 patients at 10 primary care sites in the USA (sensitivity 87.4%, specificity 89.5%), which led to it becoming the first artificial intelligence-based medical device to receive US Food and Drug Administration (FDA) approval for marketing in 2018. In 2020, The EyeArt Automated DR Detection System was the second AI-based DR grading programme to become FDA approved, after it demonstrated 95.5% sensitivity and 85% specificity in detecting referrable DR and 95.1% sensitivity and 89% specificity in detecting vision-threatening DR in a prospective multicentre study across fifteen US study centres [50]. EyeArt has also been used in combination with smartphone-based fundal photography in India to provide DR screening with minimal infrastructural requirements [51]. Code-free DL models based on automated machine learning (AutoML), a set of cloud-based tools that automates AI model development, provides prerequisite hardware, and offers an interface requiring no coding expertise, have been demonstrated to detect referrable DR from five field CFPs captured with handheld portable cameras within a regional screening programme in the Philippines with 94% sensitivity and 97% specificity on external validation [52]. Optos Plc, in partnership with Google LLC and Verily Life Sciences LLC, is seeking to bring to market its DL-based DR detection algorithm utilising images from its ultra-widefield (UWF) confocal scanning laser pseudocolour retinal imaging devices [53]. Clinical trials of this algorithm are ongoing. Real-world impact from DL-based DR screening programmes has been demonstrated in Thailand, where a prospective interventional cohort study comparing a DL-based programme developed by Google LLC [42] with retinal specialist graders within the Thai healthcare system screened 7651 diabetic patients. The DL-based programme achieved 94.7% accuracy, 91.4% sensitivity and 95.4% specificity in detecting vision-threatening DR, equivalent to the specialist graders in terms of accuracy and specificity, and statistically superior in terms of sensitivity. This study also demonstrated the challenges of implementing DL systems within low and middle-income countries, including barriers to integration within existing workflows which may be paper-based, poorer referral tracking systems, and higher proportions of ungradable images (secondary to cataract, camera operator error or faulty camera equipment) [54].

A potential concern of DL models is their limited ability to detect pathology aside from the usually single disease for which they have been trained. A Singapore-based DL algorithm, named SELENA + , has attempted to address this concern by training the model to recognise not only referrable DR but also glaucoma and age-related macular degeneration (AMD). Having been trained on almost 500,000 images, it has been reported to have 90.5% sensitivity, 91.6% specificity and 0.936 area under the receiver operator curve (AUC) in detecting referrable DR within the SiDRP, with subsequent external validation in six countries. It could simultaneously detect possible glaucoma with sensitivity 96.4% and specificity 87.2%, and AMD with sensitivity 93.2% and specificity 88.7% [55]. SELENA + has now been deployed to the entire SiDRP in a semi-automated workflow, where cases are categorised as non-referable or referrable by the algorithm, with only those identified as referrable being subsequently manually graded by human trained technicians [56].

### Retinopathy of prematurity

Retinopathy of prematurity (ROP) is a proliferative retinal vascular disease of preterm low birth weight neonates that is the leading cause of childhood blindness [57]. It is estimated to affect 68% of neonates with birth weight less than 1251 g, with more than a third of such cases classified as severe [58, 59]. Since the landmark CRYO-ROP study in 1988, ROP has become a treatable disease, with successful treatment being dependent on early recognition of sight-threatening features such as plus disease [60, 61]. Screening programmes for preterm low birth weight neonates have therefore become vital in reducing rates of blindness from ROP [62–65], and the American Academy of Pediatrics recommends screening of all infants with a birth weight of ≤ 1500 g or a gestational age of 30 weeks of less [66]. Traditional screening programmes require frequent dilated fundal examination with binocular indirect ophthalmoscopy (BIO), often with scleral indentation, by ophthalmologists with substantial subspecialty experience. Several limitations to this approach are apparent: first, there is already an insufficient paediatric ophthalmology workforce in the developed world to cope with screening demand [67], and this problem is magnified in the developing world where demand is greater and the workforce is ever smaller [68, 69]. Secondly, as preterm infant survival improves through advances in neonatal medicine, the population requiring screening is likely to continue to increase worldwide for the foreseeable future [70].

Telemedicine approaches to ROP screening overcome several of the limitations of traditional screening, and are likely to continue to play a vital role in screening programme delivery in the developing and developed world in future. Requirements for telemedicine screening include a portable neonatal fundal imaging device such as the RetCam range of cameras (Natus Medical Inc., Pleasanton, California, USA) or 3nethra Neo (Forus Health), trained camera operators, infrastructure to transfer imaging data, and a system for grading images and communicating results to clinicians. The Stanford University Network for Diagnosis of ROP (SUNDROP) initiative is an example of a successful real-world telemedicine approach to ROP screening. Based in 6 neonatal intensive care units (NICU) in California, USA, it screened 608 preterm infants over 6 years. NICU nurses were trained to capture at least 5 wide-angle CFPs per eye using the RetCam II/III. These images were transferred via secure routes to a single reading centre where ROP specialist ophthalmologists would grade the images. Grading via telemedicine was compared with a gold standard of BIO examination of the same infants by specialised paediatric ophthalmologists within one week of discharge, with remote interpretation demonstrating sensitivity of 100%, specificity of 99.8%, positive predictive value of 95.5% and negative predictive value of 100% for treatment-warranting ROP (TW-ROP) [71]. In India, the Karnataka Internet Assisted Diagnosis of ROP (KIDROP) programme trained technicians to capture and grade CFPs using the RetCam Shuttle across 36 NICUs from 2011 to 2015, screening 7106 infants. Images were also securely transferred for centralised grading by ROP experts. It found an overall ROP incidence of 22.4% and TW-ROP incidence of 3.6%. The study demonstrated the feasibility of a telemedicine screening approach in a rural environment in the developing world, and also the potential for non-expert ophthalmologists to grade images, although a significant limitation of the study was that no comparison with gold standard live BIO examination was reported [72].

A limitation of the above telemedicine programmes is the need for trained diagnosticians to grade the numerous fundal images generated by ROP screening. Automated detection of TW-ROP is a potential solution in situations where there are insufficient trained graders, a situation which may be encountered particularly in the developing world. DL algorithms to detect plus disease, a useful marker of TW-ROP, have been created to fulfil this role, with several models validated and shown to have high diagnostic accuracy [73–78]. Wagner et al. have also demonstrated the potential of utilising pre-trained CNNs to develop code-free deep learning models to detect pre-plus and plus disease [79]. Safe real-world deployment of these algorithms is yet to occur, with potential barriers including training of models on specific ethnic groups limiting their generalisability to other populations, and poor performance of algorithms on images captured by devices other than that on which they were trained [80].

### Referral refinement

Ophthalmologist workload is increasing globally, particularly in the field of retina, as a result of demographic changes and increasing prevalence of DM, together with a low number of ophthalmologists per capita: in the developed world, the population aged over 60 is growing at twice the rate of the number of ophthalmologists [81]. In addition, the increasing availability of OCT and ultra-widefield imaging in the optometrist setting has resulted in increased detection of asymptomatic retinal conditions and consequent specialist referrals [82]. In the National Health Service (NHS) in the UK, there were 7.5 million attendances to ophthalmology clinics from 2021 to 2022, the highest number for any specialty [83]. There is therefore a clear need to accurately triage referrals to hospital eye services (HES) in order to avoid unnecessary referrals and associated waste of tertiary care resources, whilst safely prioritising cases that do need hospital review according to clinical urgency. Telemedicine approaches to referral refinement have demonstrated the ability to meet this need. A real-world retrospective cohort study based in Denmark demonstrated the benefit of referral refinement through introduction of a telemedical service intermediary between community optometrists and HES: all patients whom optometrists from the largest optometrist chain in Denmark wished to refer to HES had their examination and investigation findings uploaded to a cloud-based system (Optoflow, Kide, Finland). This data was then reviewed remotely by a group of consultant ophthalmologists who determined most appropriate follow-up. Of 9938 patients referred to HES between 2018 and 2019, the telemedicine service referred only 19.5% to HES, whilst 14.4% required no follow-up and 66.1% required follow-up either with the referring optometrist or the telemedicine service [84]. In the UK, a retrospective cohort study utilised a similar pathway to refine optometrist referrals to HES for retinal disease and found that only 14% required urgent HES review, 34% required routine HES review, and 52% required no HES referral at all [85].

Referral refinement via telemedicine, with avoidance of unnecessary referrals and more timely review of urgent referrals, has clear potential benefits as discussed above. However, there is a need for improved safety data to ensure that referrals designated via telemedicine as not requiring HES review do not come to harm from incorrect triage (false negatives). In addition, the above referral refinement programmes are resource-intensive, with robust and secure cloud-computing software and networking requirements to link referrers and HES, together with dedicated ophthalmologist time to triage a significant number of referrals, which may limit its implementation in the developing world. In future, automated referral refinement may reduce manpower requirements, and video consultations with referred patients may increase accuracy and safety of triage outcomes [86].

### Portable devices

The use of portable screening devices in telemedicine has been demonstrated to be effective in early detection and triaging of chronic eye diseases [87]. Studies have also shown that early detection of such diseases is associated with favourable resource utilisation and costs [88–90]. Taylor et al. estimated that there is a five-time cost savings return to the community in terms of productivity and quality of life from primary prevention of eye disease [91]. Despite the evidence that early detection of chronic eye disease results in better outcomes, there is a lack of data on the optimal eye screening model which can be partly attributed to limitations of current diagnostic devices and models of cost-effectiveness of eye screening. In a survey of 104 family physicians in Singapore, 89 (86%) felt that teleophthalmology would reduce the number of unnecessary referrals to a specialist but cited a lack of diagnostic equipment as a major barrier to implementation [92].

## Telemedicine in the hospital setting

The ‘virtual clinic’ (VC), defined by the Royal College of Ophthalmologists, UK, as patient-clinician consultations in which the face-to-face (F2F) interaction is removed [93], is an innovation that has transformed patient care pathways over the past decade, and will continue to do so for the foreseeable future [94]. It has been made possible in the field of ophthalmology by the increasing availability and quality of diagnostic equipment, upskilling of technicians to use such equipment, methods to store and securely forward results to reviewing clinicians, and secure networked communication channels to act on findings and communicate plans to patients in a timely manner. The virtual clinic has been shown to complement, and often replace, traditional live F2F clinics in a significant proportion of patient interactions with HES, simultaneously improving efficiency of HES resource use, maintaining patient safety, and improving inclusivity and equality of care provision [95–99]. The ability of the virtual clinic to generate additional HES capacity is vital, as the number of referrals from community care providers continues to increase for the reasons mentioned in the previous section.

In the UK, VCs were first introduced in the field of glaucoma. Rathod et al. demonstrated the safety of a virtual glaucoma clinic by comparing diagnoses made from asynchronous review of nurse examination and investigations with F2F diagnoses, showing good agreement between the two methods, and 94.4% sensitivity and 86.7% specificity for diagnosis of glaucoma via the virtual pathway [100].

Retinal services are particularly suited to the VC model in light of the emergence of advanced retinal imaging methods, such as high resolution widefield and ultra-widefield colour fundus imaging and OCT, which have revolutionised and become the cornerstone of diagnosis and monitoring of retinal disease. Several examples of successful retinal VC pathways have been published, with two main models described. Most commonly, the pathway consists of patient completion of a standardised questionnaire to determine relevant history, capture of visual acuity (VA), IOP, and imaging in the virtual clinic with patients returning home thereafter, asynchronous clinician review of results, and written or telephone communication of the subsequent plan [95]. Alternatively, the same pathway is followed but with the addition of assessment for ‘red flag’ features during virtual assessment that, if present, trigger same day F2F review [99]. The potential benefits of the latter approach include increased safety by managing presentations requiring same-day intervention appropriately, and avoidance of duplicate appointments in rapid succession for such cases. However, such a pathway requires a F2F clinic to be running concurrently, and may place excess strain on an already full capacity F2F clinic with an unpredictable number of extra patients to review. VCs may be ‘single-disease’, facilitating an algorithmic approach to data capture and assessment, potentially by trained graders rather than ophthalmologists, or ‘mixed-pathology’, facilitating increased scope of suitable cases for virtual review and reduced burden on F2F clinics.

Moorfields Eye Hospital (MEH) NHS Foundation Trust has implemented virtual medical retina (MR) clinics since 2015. MEH South Division has reported outcomes of mixed-pathology virtual clinic review of retinal referrals (either external from the community or internal referrals from other ophthalmic subspecialties) triaged as low-risk, with 1729 patients reviewed between 2016 and 2017. Key findings include a 45.5% discharge rate and 37.1% continued VC follow-up rates for external referrals after first VC, with only 17.4% of patients being brought for F2F assessment subsequent to first VC review. Of those brought for F2F review, 34.7% of these were due to poor image quality and 20.2% due to the need for urgent treatment [95]. A follow-up study by the same group demonstrated that between first and second VC appointment, there was no reduction in mean VA or mean DR severity, suggesting that it is safe to keep selected patients within the VC setting without need for intermittent F2F appointments [96].

Single-disease VCs have also shown significant promise and real-world success. A randomised clinical trial of VC vs F2F screening and monitoring of neovascular AMD demonstrated that referral-to-treatment time was the same for both approaches, and although mean recurrence-to-treatment time was longer for VC-monitored patients than F2F (13.6 versus 0.04 days), this did not result in any difference in mean VA between the groups at the end of the trial [101]. Diabetic eye disease has also been shown to be amenable to the VC approach: a retrospective cohort study at MEH over a 4-year period assessed the proportion of referrals to HES from the England Diabetic Eye Screening Programme that were suited to VC review, and compared outcomes from VC versus F2F pathways. Of 12,563 newly referred patients, 70.7% were found to be suitable for VC, although only 18.4% were referred to VC due to capacity constraints. Urgent referrals were automatically directed to the F2F pathway. The mean time from routine referral to assessment was shorter for VC than F2F clinics (68 versus 80.9 days), as was the time from referral to discharge at first appointment where appropriate (71.3 versus 86.5 days). For patients requiring intravitreal injection therapy, there was no statistically significant difference in mean time from referral to first injection. The study authors conclude that VC review of appropriately selected DR referrals improves efficiency of MR services without compromising patient safety [97].

The COVID-19 pandemic has emphasised the strengths of the VC model and accelerated its adoption [102]. The high throughput of VCs helps to address the significant backlog of referrals resulting from delayed presentation and reduced clinic capacity during the pandemic. Predictable and streamlined patient flow with shorter patient journeys within VCs than F2F clinics has facilitated social distancing within waiting areas [99]. Reduced F2F interaction time in the VC facilitates reduced time for potential transmission of airborne diseases between patients and staff.

Despite strong evidence of the benefits of the VC pathway to HES and patients, there remain barriers to its widespread adoption. A major limitation is the high initial outlay in facilities, diagnostic equipment, secure networked data storage and transfer solutions meeting information governance requirements, and hiring and training of technicians required to set up a high-capacity VC system. In healthcare systems where commercial or state reimbursement are the primary source of HES funds, this problem can be exacerbated by reduced reimbursement for virtual versus F2F appointments [103]. These set-up costs, together with requirements for robust and secure telecommunication infrastructure, may particularly limit the adoption of the VC in developing nations, risking accentuation of the healthcare divide between the developed and developing world [104]. In addition, there are safety concerns associated with the VC pathway. There is the risk that patients attending the VC who require same-day intervention will have their treatment delayed due to asynchronous review of results, although appropriate implementation of a red flag pathway during VC patient assessment may reduce this risk [99]. Diagnoses outside of the remit of the VC may be missed, although this is also possible in F2F clinics. The patient perspective is vitally important, and the potential loss of live doctor-patient interaction, a cornerstone of the doctor-patient relationship, may detrimentally affect VC care outcomes [105]. Further research on maintaining safety, reducing costs, and ensuring patient acceptance of and satisfaction from the VC pathway should help improve its future uptake within the field of retina.

## Teleophthalmology and the patient experience

High patient satisfaction with synchronous telemedicine in ophthalmology, especially over the course of the COVID-19 pandemic, has been widely reported [106–109]. Studies have also highlighted positive provider attitudes towards teleophthalmology and its continued use [110–113]. This is unsurprising given the numerous benefits of teleophthalmology to both patients and healthcare providers [114] (Table 2). Nonetheless, there remain several disadvantages and barriers to teleophthalmology faced by patients (Table 2). This might account for the hesitance among certain patient groups (e.g. older age, lower educational attainment, telehealth inexperience) towards its adoption [115].

**Table 2:** Benefits and disadvantages/barriers of teleophthalmology for patients

Benefits	Comment
Large outreach	Overcomes geographical barriers to healthcare access [116, 117]
Timely evaluation and access to emergency intervention	Addresses coverage gap for emergency eye care and prevent delay in initiation of treatment [118]
Avoiding unnecessary referrals	Proper triaging to select patients who require intervention [e.g. most patients referred for fundal examination by primary care did not require treatment [119]]
Saves cost, time and effort	Teleophthalmology for screening of retinal diseases such as diabetic retinopathy has been shown to be cost-effective [120]
Infection control and protection	Minimises avoidable patient contact, especially for those at a high risk of communicable disease [121]
Comparable diagnostic accuracy	Teleophthalmology consultations have high agreement with face-to-face consultations in the diagnosis of retinal conditions such as AMD [122]

Disadvantages/Barriers	Comment
Unfamiliarity	Patients may struggle to access telemedicine due to low digital literacy [123]
Poor image quality	Media opacities (e.g. cataract or vitreous haemorrhage) or poor compliance can lead to inadequate and ungradable fundal images [124]
Privacy and security concerns	Inadvertent transmission of non-clinical information or sharing of data with third-party advertisers may cause patients to lose trust [125]
Patient-doctor relationship	Concerns exist about depersonalisation of the patient-doctor relationship due to a lack of physical interaction which might affect trust and communication [126]

Given the emerging and increasing popularity of virtual clinics within secondary care for high-volume outpatient ophthalmic subspecialties such as medical retina to address capacity issues [99, 127], there is a need to assess and define patient suitability (Table 3) for such services to enhance patient experience and maintain patient safety. Recent evidence from other subspecialties such as glaucoma suggests that virtual clinics with expanded eligibility criteria can remain effective in delivering high-quality, safe care with high levels of patient satisfaction [128].

**Table 3:** Patient factors determining suitability for virtual clinics

Factors	Clinic type	
	Virtual	Face-to-face
Risk of infectious disease (e.g. COVID-19)	High	Low
Digital proficiency	High	Low
Clinical urgency	Low	High
Risk of delaying clinical procedure / surgery	Low	High
Vulnerability	Low	High
Communication difficulties (e.g. dementia)	No	Yes

Several sociodemographic factors such as older age, Asian ethnicity and non-English speakers have been identified to be associated with lower rates of telemedicine use [129]. At MEH, male gender, socioeconomic deprivation, previous appointment cancellation, and lack of self-reporting of ethnicity were found to be associated with non-attendance in synchronous audiovisual appointments [130]. Lack of resources, digital literacy and trust in the video consultation model have also been found to contribute to the “digital divide or exclusion” in teleophthalmology [131, 132], which risks compounding existing health disparities. Clear guidelines and strategies are required to address and minimise such inequalities in access to enhance digital healthcare provision.

## Teleophthalmology and the doctor experience

Provision of high quality and high volume telemedicine requires appropriate information technology (IT) infrastructure which include high-speed internet connectivity, up-to-date imaging software and continuous access to technical support [133]. While there is usually a significant amount of time and resources incurred with its initial implementation, adequate IT infrastructure investment has been shown to be more cost-effective in the long run from a global perspective [134, 135].

Interprofessional communication can be enhanced by teleophthalmology through rapid access to specialist advice from any location [117]. Various modes of consultation currently exist (Table 4) and secure messaging apps (e.g. Pando, Slack, Induction Switch) allow users to connect with colleagues for expert advice and support, thus improving patient care. Efficient triage and decision can also be made by ophthalmologists following review of relevant patient history and retinal imaging via a cloud-based referral platform. This has been shown to yield time-efficient primary care referrals and reduce unnecessary tertiary retinal referrals within hospital services [85].

**Table 4:** Various modes of teleconsultation

Mode	Platform example	Advantages	Disadvantages
Video	Attend anywhere [136, 137]	• Closest to face-to-face consultation • Offers important visual information • Better patient rapport • More reliable patient identification	• Requires more complex and expensive setup (i.e. high-quality webcam and internet connection) • Vulnerable to privacy and security risks
Phone/Audio	Digital enhanced cordless telecommunications (DECT) phones [138]	• Convenient and quick • Cheaper given less IT infrastructure requirement • Widely accessible	• No visual cue(s) • May be difficult to establish patient rapport
Text-based	Florence [139]	• Convenient and quick • Cheapest given minimal IT infrastructure requirement • Widely accessible	• Communication is largely asynchronous • No audio or visual cue(s) • Difficult to establish patient rapport
Hybrid	N/A—consists of alternating face-to-face and virtual consultations [140, 141]	• Screening/diagnostic tests, physical examination and treatments can be incorporated • More efficient care pathways may be created	• More costly • Gap in accessibility among those with digital literacy challenges

A survey of UK oculoplastic surgeons in 2020 demonstrated increased utility of and confidence in telemedicine in routine clinical practice, and improved quality of telemedicine infrastructure, as a consequence of the COVID-19 pandemic. Improvement of patient flow from telemedicine adoption, with reduced time from referral to first clinic appointment, improved efficiency of service provision, and low recall from telemedicine consultations for face-to-face assessment, was noted by the majority of respondents. Barriers to telemedicine integration highlighted by clinicians included difficult patient examination, limitation in rapport-building, lack of administrative support and poor patient access to digital technology [113].

## Home monitoring

Home monitoring via patient self-measurements has been shown to be a valuable adjunct of modern teleophthalmology [142, 143]. In addition to reducing the need for clinical visits, it facilitates the collection of high quality data in a personal setting that can guide targeted management [143]. An example of a widely used, long-established method for this is the Amsler grid which helps to detect signs of visual deficits such as metamorphopsia in patients with macular diseases [144]. Although such traditional methods may be useful in disease screening, it is not well suited for monitoring of disease progression given its imprecise and unquantifiable assessment of visual function [145].

In an attempt to address this gap, the recent years have seen the introduction of a myriad of digital home measurement tests [146]. These include home-based and smartphones/tablets-based devices which have been shown to be cost-effective in specific patient cohorts [147]. An example of the former is the ForeseeHome (Notal Vision Inc., Manassas, VA, USA), a home-based digital hyperacuity testing device for patients with age-related macular degeneration (AMD) that transmits data directly to an ophthalmologist [148]. The clinical utility of the ForeseeHome system was demonstrated by a randomised controlled trial which had to be terminated earlier due to its superior efficacy at an interim data analysis [148]. However, real-world evidence has since demonstrated a significant proportion of false-positive alerts with this device [149]. Nonetheless, these alerts have been found to be highly predictive for future conversions in high-risk eyes that are fellow to eyes with neovascular AMD [150].

Miniaturisation of optical coherence tomography (OCT) imaging technology for home monitoring has also been of particular interest [151, 152]. An example of this is the Notal Vision Home OCT (NVHO, Notal Vision Inc.) system which has demonstrated feasibility in two recent prospective longitudinal studies [152, 153]. Using a validated artificial intelligence-based software that detects and quantifies retinal fluid on OCT [154, 155], automated analysis of self-acquired scans in patients with neovascular AMD achieved a high agreement with human grading. A large multicentre randomised clinical trial in which home OCT-guided treatment versus treat and extend for the management of neovascular AMD is currently being planned (DRCR.net Protocol AO: https://public.jaeb.org/drcrnet/view/UpcomeStudies). Other available options of home-based OCT include the self-examination low-cost full-field OCT (SELFF-OCT) currently patented and commercialised by Visotec (https://visotec.health/) [156, 157], and the sparse OCT (also known as MIMO-OCT) developed by a Swiss group [158].

Despite the promising role of home-based OCT, its high upfront hardware cost, coupled with the ever-increasing incidence of degenerative macular pathology with an ageing population, is likely to limit its reachability in the near future [146]. More readily available smartphones or tablets that can administer remote tests of visual function have a greater potential to maximise patient outreach. Examples of currently available mobile apps that implement novel tests include Alleye [159] and Home Vision Monitor (previously known as myVisionTrack^®^) [160], both of which are FDA-cleared and Conformité Européenne (CE) marked. Alleye employs a monocular alignment hyperacuity task [159] whereas Home Vision Monitor utilises a shape discrimination hyperacuity test. Patient acceptability, validity and real-world utility of both apps have been examined by several studies with encouraging results to date [143, 161–166].

## Teleophthalmology to support rural and remote communities

Rural and remote communities, especially in large countries, often consist of small but widely dispersed populations and represent a major hurdle to healthcare access [167]. Teleophthalmology provides a unique opportunity to address this disparity by facilitating timely and wider access to healthcare. Although this has been implemented for various retinal conditions, diabetic retinopathy (DR) is the most targeted disease given that it remains the leading cause of preventable blindness in working-age adults worldwide [168]. There is also evidence to suggest that rural populations are disproportionately affected by DR [169].

Several teleophthalmology programs for rural communities have been established across the globe [170–174]. However, the uptake and implantation of teleophthalmology in remote services remain underwhelming despite its vast potential in promoting equity and accessibility to eye care. The reasons for this are multifactorial and revolve around the logistical challenges in coordinating outreach optometry and ophthalmology services as well as ensuring availability of dedicated on-call specialists [116].

DL systems may present a unique solution to the barriers faced by teleophthalmology in rural areas [175]. Effective triage and assessment of patients with retinal diseases via such systems can overcome the main hurdle of accessing an ophthalmologist in remote areas. With the continuous refinement of DL systems, AI-augmented teleophthalmology appears poised to make remote retinal screening more automated, timely and cost-effective [176]. The implementation of 5G networks may even allow novel models of remote treatment delivery such as “telephotocoagulation” which involves creating image-based and fluorescein angiogram-based treatment plans for remote navigated retinal photocoagulation [177].

## Teleophthalmology and the COVID-19 pandemic

The COVID-19 pandemic saw an exponential growth in teleophthalmology with the expansion of existing services due to the mandatory policies of self-isolation and social distancing to maintain patient and clinician safety. Given that the traditional face-to-face patient-physician model alone was no longer fit for purpose, rapid deployment of digital technology and new models of care became necessary to meet the growing demand and expectations. Various digital health operational models have since been adopted, either independently or in combination, to facilitate new processes for clinical care [121].

Within ophthalmology, patients with stable retinal conditions not requiring active intervention have been recommended to adopt remote care through virtual clinics [99, 178] and home monitoring [161], while vulnerable and high-risk patients continued to attend face-to-face visits. Furthermore, the goal of care had to be re-evaluated for patients with specific retinal diseases. An example of this is neovascular AMD, in which patients were recommended fixed-dosing and treat-and extend regimes of anti-VEGF injections to reduce the need for non-treatment follow-up visits [179, 180]. This creates a situation where all face-to-face visits are for anti-VEGF treatment, thus obviating the need for traditional monitoring visits.

The provision of emergency ophthalmic care via teleophthalmology has also been shown to be feasible and effective [136, 137]. An exemplar of this is the Moorfields virtual eye casualty service that was launched out of necessity during the COVID-19 pandemic [136]. This provided video consultations directly between patients and ophthalmologists, allowing drop-in access without a referral as well as effective triaging of patients (i.e. out-of-hospital management of low-risk patients and selective signposting of high-risk patients to a less crowded A&E for traditional care). Overall, this novel service utilising videoconsultation was found to be acceptable by patients and had comparable patient safety to traditional in person review [137].

## Conclusion

The integration of telemedicine within ophthalmology and in particular the field of retina has already transformed the way in which healthcare is delivered, both in the community and in the hospital setting. A myriad of factors including ever-increasing demand for services, improvements in global telecommunications infrastructure, increasing computing power and the advent of AI have made the approach increasingly viable, with the COVID-19 pandemic providing the catalyst for its widespread acceptance and adoption across the globe.

With the exponential development and integration of technological innovations complementary to digital health such as deep learning and the ‘Internet of things’, it is reasonable to envisage a future where teleophthalmology will be at the forefront of eyecare transformation by facilitating automation of clinical decision making with a data-driven approach as well as personalised eye care through continuous personal data monitoring and analysis.

Given these ongoing developments, it does not seem far-fetched to think that we are at the beginning of a golden age of teleophthalmology. Nevertheless, much of the current body of evidence in the field is limited to specific domains such as population screening, and more work is needed in terms of robust randomised controlled trials and health economic analyses to broaden the application, adoption, and acceptability of teleophthalmology in a wider global context. Other key challenges at a higher organisational level include the lack of tools and capabilities by commissioning services to drive change as well as the lack of a leadership culture and organisational infrastructure to support this. Patient engagement in steering planning and satisfaction assessment from early deployment of teleophthalmology programmes also remains vital, and is especially pertinent in light of the growing importance of patient-led care.

As we continue to witness a paradigm shift in how ophthalmic healthcare is delivered, it is vital that these challenges are addressed. Teleophthalmology systems must ultimately remain safe, validated, secure, and include measures to reduce digital exclusion and the exacerbation of existing health inequalities, both within nations and between the developed and developing world. With these safeguards in place, the present and future benefits of telemedicine to patients and physicians are unparalleled.

## Data Availability

Not applicable.
